# LncRNA‐SNHG15 enhances cell proliferation in colorectal cancer by inhibiting miR‐338‐3p

**DOI:** 10.1002/cam4.2105

**Published:** 2019-04-03

**Authors:** Min Li, Zehua Bian, Guoying Jin, Jia Zhang, Surui Yao, Yuyang Feng, Xue Wang, Yuan Yin, Bojian Fei, Qingjun You, Zhaohui Huang

**Affiliations:** ^1^ Wuxi Cancer Institute Affiliated Hospital of Jiangnan University Wuxi Jiangsu China; ^2^ Cancer Epigenetics Program Wuxi School of Medicine Jiangnan University Wuxi Jiangsu China; ^3^ Pharmacy Department Wuxi 9th People's Hospital Affiliated to Soochow University Wuxi Jiangsu China; ^4^ Department of Surgical Oncology Affiliated Hospital of Jiangnan University Wuxi Jiangsu China

**Keywords:** colorectal cancer, FOS, long noncoding RNA, miR‐338‐3p, RAB14, small nucleolar RNA host gene 15

## Abstract

The incidence and death rate of colorectal cancer (CRC) is very high, which brings great need to understand the early molecular events of CRC. These studies demonstrate that long noncoding RNA (lncRNA) plays an important role in the occurrence and development of human cancer. Small nucleolar RNA host gene 15 (SNHG15) was recently identified as a cancer‐related lncRNA. In this study, we aimed to evaluate the function and mechanism of SNHG15 in CRC. The expression of SNHG15 was detected by quantitative RT‐PCR (qRT‐PCR) in CRC tissues and matched noncancerous tissues (NCTs). CCK‐8 assay, colony formation assay, flow cytometric analysis, and nude mouse xenograft mode were used to examine the tumor‐promoting function of SNHG15 in vitro and in vivo. The binding relationship between SNHG15, miR‐338‐3p and the target genes of miR‐338‐3p were screened and identified by databases, qRT‐PCR, dual luciferase reporter assay and western blot. Our results showed that SNHG15 was up‐regulated in CRC tissues compared with paired NCTs (*P* < 0.0001). High level of SNHG15 expression predicted poor prognosis of CRC (*P* = 0.0051). SNHG15 overexpression could promote cell proliferation and inhibit cell apoptosis. Animal experiments showed that up‐regulation of SNHG15 promoted tumor growth in vivo. The results of mechanism experiments showed that SNHG15 could bind to miR‐338‐3p and block its inhibition on the expression and activity of FOS or RAB14. In conclusion SNHG15 promotes cell proliferation through SNHG15/miR‐338‐3p/FOS‐RAB14 axis in CRC.

## 1 INTRODUCTION

Colorectal cancer (CRC) is one of the most common malignant tumors in the world, and it is one of the leading causes of cancer‐related death worldwide.^1^, ^2^, ^3^ The occurrence and development of CRC is a complex process and involves a series of genetic and phenotypic changes.^4^, ^5^ The molecular mechanism underlying the occurrence and development of CRC is not clear yet, recent studies have shown that long noncoding RNA (lncRNA) plays an important role in the occurrence and development of CRC.

Studies have indicated that noncoding RNAs (ncRNAs) account for nearly 98% of the human genome transcripts, and lncRNA is one of the most common studied ncRNAs.^6^, ^7^, ^8^ LncRNA is a kind of endogenous transcript with more than 200 nucleotides in length, and lacking specific and complete open reading frame.^9^ LncRNA has been confirmed to be associated with many human diseases, especially with tumors. A large number of studies have reported that lncRNA plays an extremely important role in the occurrence and development of human cancers, including CRC.^10^ For example, HOTAIR promoted cell proliferation and chemoresistance of CRC via miR‐203a‐3p‐mediated Wnt/ß‐Catenin signaling pathway.^11^ LncRNA‐H19 accelerated cell proliferation and metastasis in CRC and melanoma cells.^12^, ^13^, ^14^ Our previous studies demonstrated that UCA1, LINC00152, FEZF1‐AS1, and SNHG6 function as oncogenes to promote cell proliferation, metastasis, and chemoresistance of CRC cells.^15^, ^16^, ^17^, ^18^

Small nuclear RNA host gene 15 (SNHG15) is a recently identified lncRNA and locates in chromosome 7p13. Researches showed that SNHG15 is overexpressed in gastric cancer (GC), nonsmall cell lung cancer (NSCLC) and breast cancer, and it could promote cell proliferation in these cancers.^19^, ^20^, ^21^, ^22^, ^23^, ^24^ In this study, we proved that SNHG15 is up‐regulated in CRC tissues than the matched noncancerous tissues (NCTs). The overexpression of SNHG15 indicated poor prognosis of CRC patients. Functional experiment demonstrated that SNHG15 enhances cell proliferation and inhibits cell apoptosis of CRC. Mechanistic studies revealed that SNHG15 promotes CRC tumorigenesis and progression by binding to miR‐338‐3p and impairing its inhibitory effect on the targets (FOS and RAB14). This study provides a new evidence of the regulatory network of SNHG15, miR‐338‐3p and FOS/RAB14 in CRC.

## 2 MATERIALS AND METHODS

### 2.1 Patients and tissue samples

All human primary CRC tissues and their paired adjacent NCTs used in this study were collected at Affiliated Hospital of Jiangnan University (n = 113) and Fudan University Shanghai Cancer Center (n = 90) with written informed consent. After tumor resection, tissue specimens were immediately snap‐frozen in liquid nitrogen and then stored at −80°C. This study was approved by the Medical Ethics Committees of Affiliated Hospital of Jiangnan University and Fudan University Shanghai Cancer Center. The details of study populations have been listed in Table S1. In addition, the expression profile of 479 CRC tissues and 42 NCTs were downloaded from TCGA (https://cancergenome.nih.gov/).

### 2.2 Cell lines and cell culture

HCT116, SW620, LoVo, SW480, and 293T cells were purchased from American Type Culture Collection (ATCC). SW620 cell line was cultured in RPMI 1640 medium and the other cell lines were cultured in DMEM medium containing 10% fetal bovine serum. All the cell lines were incubated at 37°C in 5% CO_2._

### 2.3 Total RNA extraction and cDNA synthesis

Total RNA was extracted from frozen tissues using RNAiso Plus (TaKaRa, Japan), and stored immediately at −80°C. Total RNA was reversed transcribed into complementary DNA (cDNA) using the PrimeScript TM II 1st Strand Synthesis Kit (TaKaRa) according to the manufacturer's instructions.

### 2.4 Quantitative RT‐PCR (qRT‐PCR) assay

The expression level of SNHG15 and target genes of miR‐338‐3p was detected by qRT‐PCR using the UltraSYBR Mixture (CWBIO, China). The sequences of primer were listed in Table S2.

### 2.5 Plasmid constructs and siRNA

The pLKD‐CMV‐G&PR‐U6‐shSNHG15 (shSNHG15) vector was purchased from OBiO (China). The SNHG15 sequence was synthesized and subcloned into the pcDNA3.1 (eukaryotic expression vector) and pWPXL (lentiviral expression vector). The 3′UTRs of potential miR‐338‐3p target genes (MAFB, FOS, RAB14, and PTEN) were amplified from genomic DNA using PrimerSTAR Premis (TaKaRa) and cloned into the luciferase reporter vector pLuc. The mutant 3′UTRs of FOS and RAB14 was constructed by overlap extension PCR and also cloned into pLuc. The siRNA of SNHG15, FOS and RAB14 were purchased from Gene Pharma (China).

### 2.6 Lentivirus production and transduction

The pLKD‐CMV‐G&PR‐U6‐shControl (shControl), shSNHG15, pWPXL, or pWPXL‐SNHG15 plasmid was cotransfected into 293T cells with psPAX2 (packaging plasmid) and pMD2G (envelope plasmid) using lipofectamine 2000 (Invitrogen, USA). Virus particles were harvested 48 hours after cotransfection and used to infect CRC cells to construct stable expression cell lines.

### 2.7 CCK8 assay, colony formation assay, and cell apoptosis analysis

Cell proliferation ability was assessed by Cell Counting Kit 8 (CCK‐8, Beyotime, China) and colony formation assay. CCK‐8 assay was conducted according to the manufacturer's instructions. For the colony formation assay, CRC cells were plated in 6‐well plate and incubated at 37°C for 2 weeks. The cells were then fixed in methanol, and stained with 0.1% crystal violet. For cell apoptosis analysis, CRC cell apoptosis was induced by 5‐FU (2 μg/mL) for 24 hours and checked using the Annexin V‐PE/7‐ADD Apoptosis Detection Kit (CWBIO).

### 2.8 Xenograft tumor assay

Ten nude mice (BALB/c) at 5 weeks of age were randomly divided into 2 groups. Totally 1 × 10^6^ CRC cells stably expressing shSNHG15, SNHG15 or control vector were subcutaneously injected into either flank of the nude mouse. The tumor formation was observed every 3 days and the tumor size was measured. After 21 days, the nude mouse was killed and the weight of the tumor was weighed. All the animal experiments were approved by the Clinical Research Ethics Committees of Affiliated Hospital of Jiangnan University.

### 2.9 Dual luciferase reporter gene assay

293T cells were cotransfected with luciferase reporter vector, microRNA (miRNA) mimic (or NC) and pRL‐CMV. The luciferase activities of these cells were analyzed using the Dual‐Luciferase^®^ Reporter Assay System (Beyotime).

### 2.10 Western blotting

Total protein of HCT116 cells transfected with SNHG15, si‐SNHG15, miR‐338‐3p, or anti‐miR‐338‐3p was extracted. Then the protein was separated by SDS‐PAGE and transferred to a PVDF membrane. The levels of FOS and RAB14 were analyzed using antihuman FOS (proteintech, 1:1000) and RAB14 (BBI Life Sciences, 1:2000) antibody. β‐actin (Beyotime, 1:1000) was used as a loading control.

### 2.11 Statistical analyses

Statistical analyses were performed using SPSS 20.0 software (SPSS, USA). All data are showed as the group mean ± standard deviation (SD). The significant differences between groups were evaluated by 2‐tailed Student's *t* test. The differences in survival rates were determined by the Kaplan‐Meier method and compared by the log‐rank test. *P* value <0.05 was considered statistically significant.

## 3 RESULTS

### 3.1 SNHG15 is up‐regulated in CRC and associated with poor prognosis

**Figure 1:**
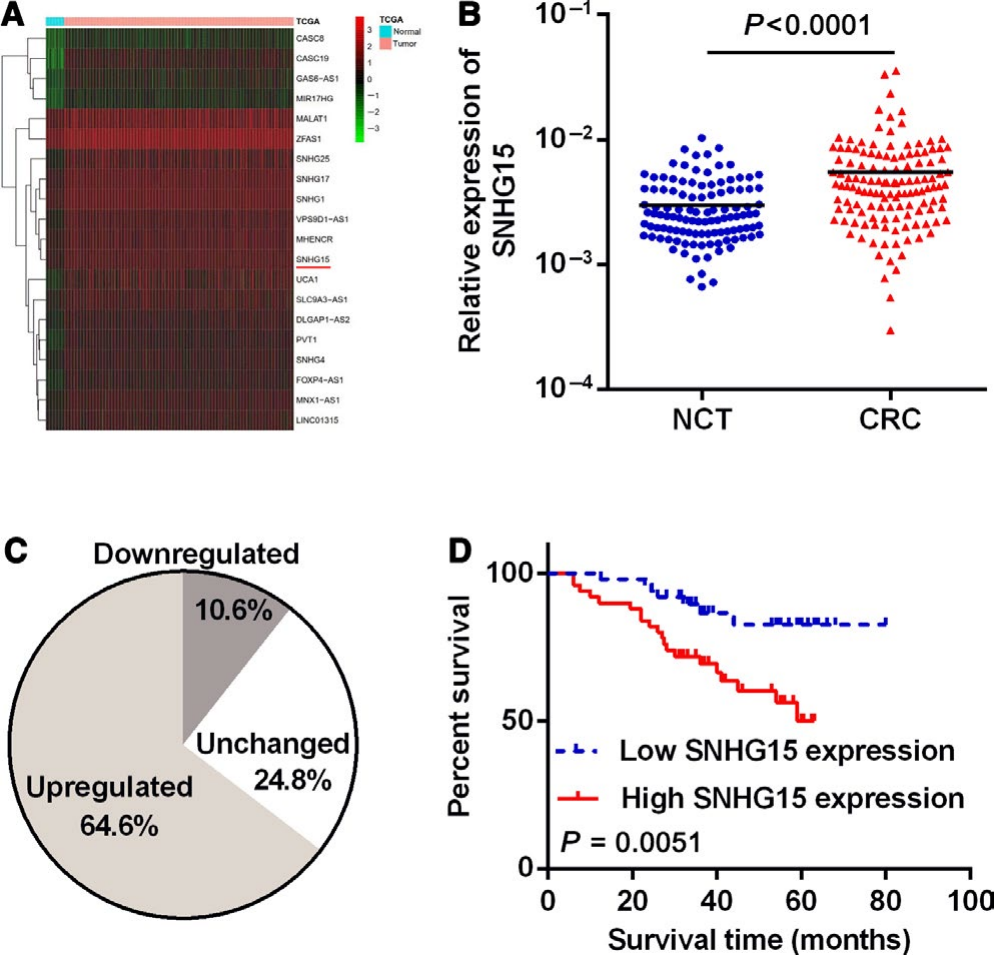
Relative expression of SNHG15 in CRC tissues. A, Relative expression levels of SNHG15 in TCGA database. B, Relative expression of SNHG15 in CRC tissues and NCTs, n = 113, *P* < 0.0001. C, The percentage of up‐regulated, down‐regulated, and unchanged SNHG15 in CRC tissues compared with paired NCTs. D, Survival analysis of CRC patients with relatively high or low level of SNHG15. *P* = 0.0051

To identify the expression level of SNHG15 in CRC, we first analyzed the expression levels of lncRNAs in CRC tissues and NCTs by analyzing TCGA database. We found that SNHG15 is one of the most up‐regulated lncRNAs among the 100 overexpressed lncRNAs (Figure 1A). Next, we applied qRT‐PCR to detect the expression of SNHG15 in CRC tissues and their matched NCTs. The results indicated that SNHG15 was significantly up‐regulated in CRC tissues (*P* < 0.0001, Figure 1B). Approximately 64.6% CRCs (73 of 113) showed more than 1.5‐fold up‐regulation of SNHG15 compared with paired NCTs (Figure 1C). Meanwhile, in order to investigate the correlation between SNHG15 expression and patients survival, we divided the CRC patients into 2 groups based on the median expression of SNHG15 in CRCs. Results revealed that increased SNHG15 expression in CRC tissues was significantly associated with a lower rate of survival (Figure 1D). These observations indicated that SNHG15 may be involved in the development and progression of CRC.

To confirm the prognostic value of SNHG15 in CRC, an independent cohort was recruited as a test group for further expression and survival analysis. The results showed that SNHG15 was also up‐regulated in this CRC cohort, and the overexpression of SNHG15 indicated poor survival for these CRC patients (Figure S1A‐C).

### 3.2 SNHG15 enhances CRC cell proliferation in vitro and in vivo

**Figure 2:**
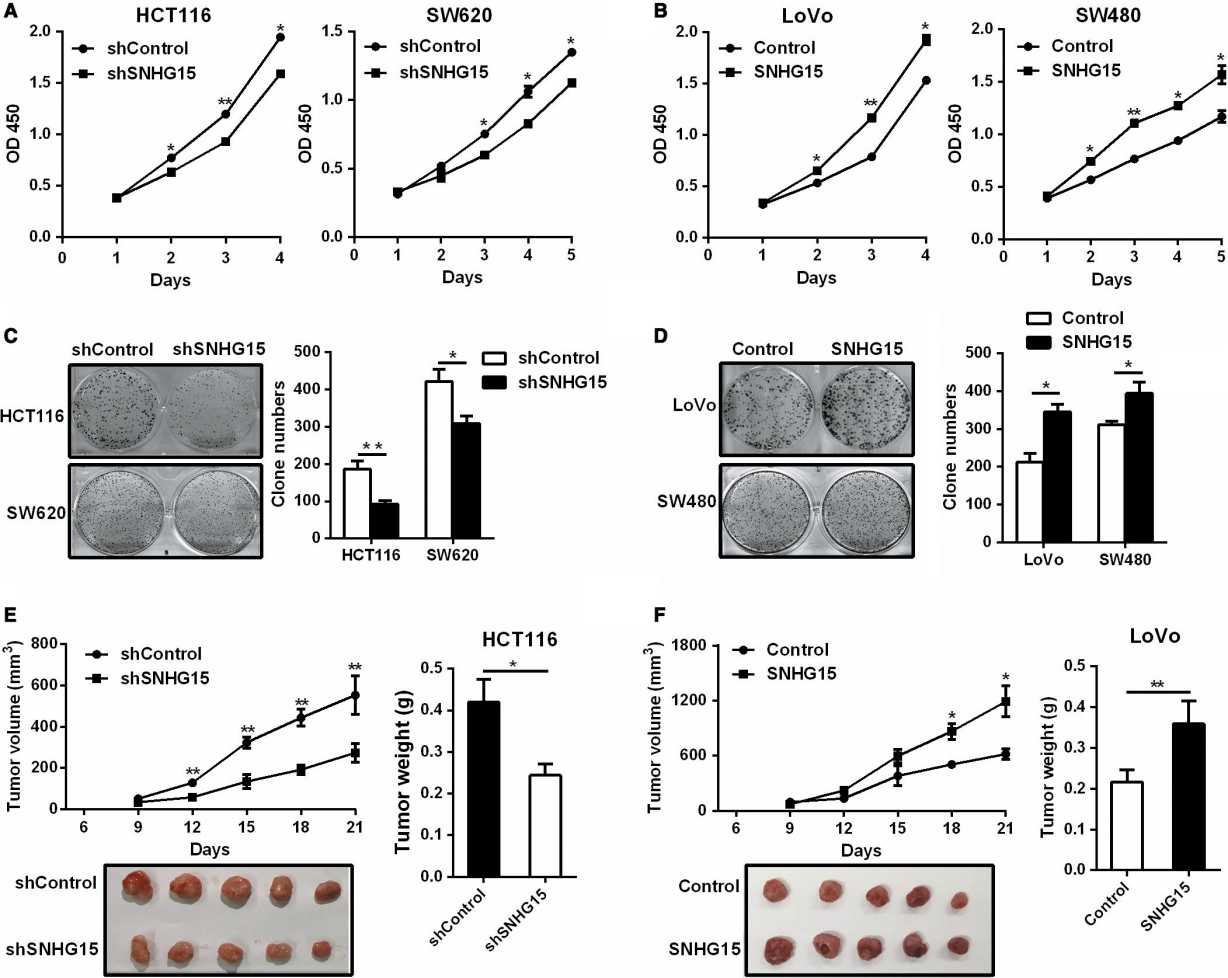
SNHG15 promoted CRC cell proliferation in vitro and in vivo. A‐B, The effect of SNHG15 knockdown (A) or overexpression (B) on cell proliferation was measured by the CCK‐8 assay. C‐D, The effect of SNHG15 knockdown (C) or overexpression (D) on cell proliferation was checked by the colony formation assay. E‐F, SNHG15 knockdown inhibited tumor growth in vivo (E); SNHG15 overexpression promoted tumor growth in vivo (F). The tumor volume were recorded every 3 days until day 21, the tumor weight were measured after sacrifice. *P < 0.05, **P < 0.01

In order to explore the function of SNHG15 in CRC, the expression of SNHG15 in 4 CRC cell lines was examined first (Figure S2A). We then knocked down SNHG15 expression in HCT116 and SW620 cells with relatively high expression level of SNHG15, and increased SNHG15 expression in LoVo and SW480 cells with relatively low SNHG15 expression (Figure S2B and C). The results showed that knockdown of SNHG15 significantly inhibited the growth of HCT116 and SW620 cells (Figure 2A). On the contrary, Up‐regulation of SNHG15 markedly promoted cell proliferation in LoVo and SW480 cells (Figure 2B). Similarly, SNHG15 could also promote the ability of colony formation in CRC cells (Figure 2C and D).

We next investigated the role of SNHG15 in CRC tumorigenesis in vivo. As showed in Figure 2E, SNHG15‐silenced HCT116 cells exhibited generally smaller tumor volume and displayed less weight compared to the control group (Figure 2E). On the contrary, LoVo cells with SNHG15 stably overexpressed obviously promoted tumor growth in vivo (Figure 2F). Meanwhile, the expression of Ki67 was also down‐regulated in the SNHG15‐depleted tumors and was up‐regulated in the SNHG15‐overexpressing tumors (Figure S3). In conclusion, these results indicated that SNHG15 could promote CRC tumor growth both in vitro and in vivo.

**Figure 3:**
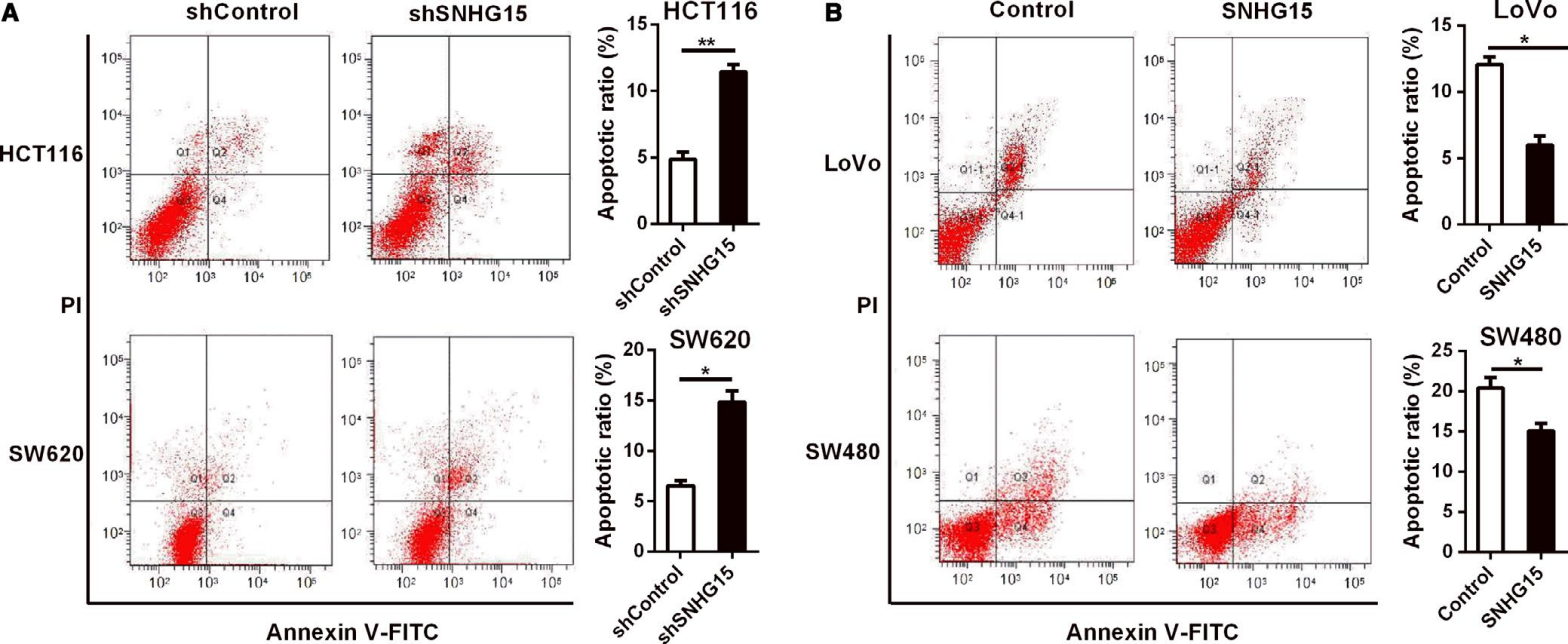
SNHG15 inhibited CRC cell apoptosis. A‐B, The effects of SNHG15 knockdown or overexpression on cell apoptosis in CRC cells were measured by flow cytometry. *P < 0.05, **P < 0.01

We also examined the effect of SNHG15 on cell cycle and cell apoptosis in CRC cells, the results showed that knockdown of SNHG15 significantly promoted cell apoptosis, whereas overexpression of SNHG15 inhibited cell apoptosis (Figure 3A and B). No obvious effect of SNHG15 on cell cycle was observed in CRC cells (Figure S4). These data indicated that SNHG15 could promote CRC cell proliferation through inhibiting cell apoptosis.

### 3.3 SNHG15 can directly interact with miR‐338‐3p

**Figure 4:**
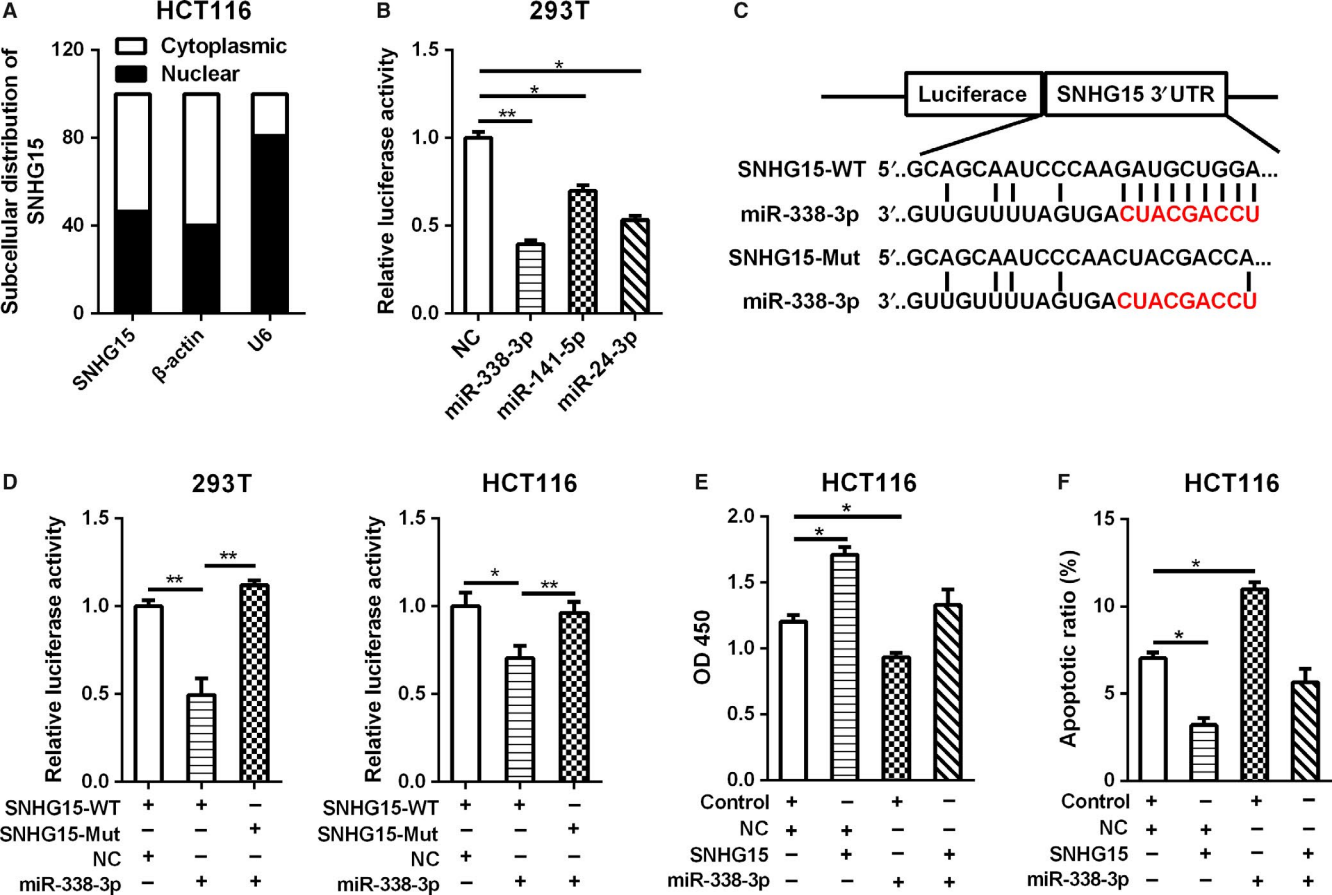
SNHG15 could directly interact with miR‐338‐3p. A, The subcellular location of SNHG15 was determined by qRT‐PCR in HCT116 cells. B, The luciferase activity of pLuc‐SNHG15 after cotransfected with miRNAs was determined by the dual luciferase assay. C, The wild type or mutant sequence of SNHG15 binding with miR‐338‐3p was cloned into the pLuc vector separately. D, The luciferase activity of pLuc‐SNHG15‐WT or pLuc‐SNHG15‐mut after cotransfected with miR‐338‐3p was determined by the dual luciferase assay in 293T and HCT116 cells. E‐F, The effect of SNHG15 on cell proliferation (E) and apoptosis (F) of miR‐338‐3p was measured by CCK‐8 assay and flow cytometric analysis. *P < 0.05, **P < 0.01

In order to explore the underlying mechanism of SNHG15 in CRC, we measured the subcellular location of SNHG15 in HCT116 cells, and found that SNHG15 was mainly located in cytoplasm (Figure 4A). More and more researches showed that cytoplasmic lncRNAs usually act as competing endogenous RNAs (ceRNAs) to regulate the binding of endogenous miRNAs to their target mRNAs and inhibit the expression of these target mRNAs. So we searched for miRNAs that might bind to SNHG15 using LNCipedia、Linc2Go、lncRNAdb and Noncode databases. The results indicated that miR‐338‐3p, miR‐141‐5p and miR‐24‐3p are most likely to combine with SNHG15. Subsequently, we confirmed that miR‐338‐3p, miR‐141‐5p and miR‐24‐3p could all bind to SNHG15 through luciferase assays, whereas miR‐338‐3p has the strongest inhibitory effect on SNHG15 (Figure 4B). To further verify whether SNHG15 could bind to miR‐338‐3p, we constructed luciferase reporter vectors of SNHG15 containing wild‐type (WT) or mutated (Mut) miR‐338‐3p binding sites (Figure 4C). The luciferase reporter assays revealed that cotransfection of miR‐338‐3p with SNHG15‐WT substantially inhibited luciferase activity of SNHG15, but miR‐338‐3p could not affect the luciferase activity of SNHG15‐Mut (Figure 4D), which indicated that SNHG15 could directly interact with miR‐338‐3p. Next, we checked the effect of SNHG15 on cell proliferation and apoptosis in miR‐338‐3p overexpressed HCT116 cells by CCK‐8 assay and flow cytometric analysis. The results showed that overexpression of SNHG15 significantly restored the inhibitory effect of miR‐338‐3p on cell proliferation (Figure 4E). The increased cell apoptosis in miR‐338‐3p up‐regulated CRC cells was also reversed by SNHG15 overexpression (Figure 4F). These results indicated that SNHG15 could directly interact with miR‐338‐3p and inhibit its function.

### 3.4 FOS and RAB14 are the target genes of miR‐338‐3p

**Figure 5:**
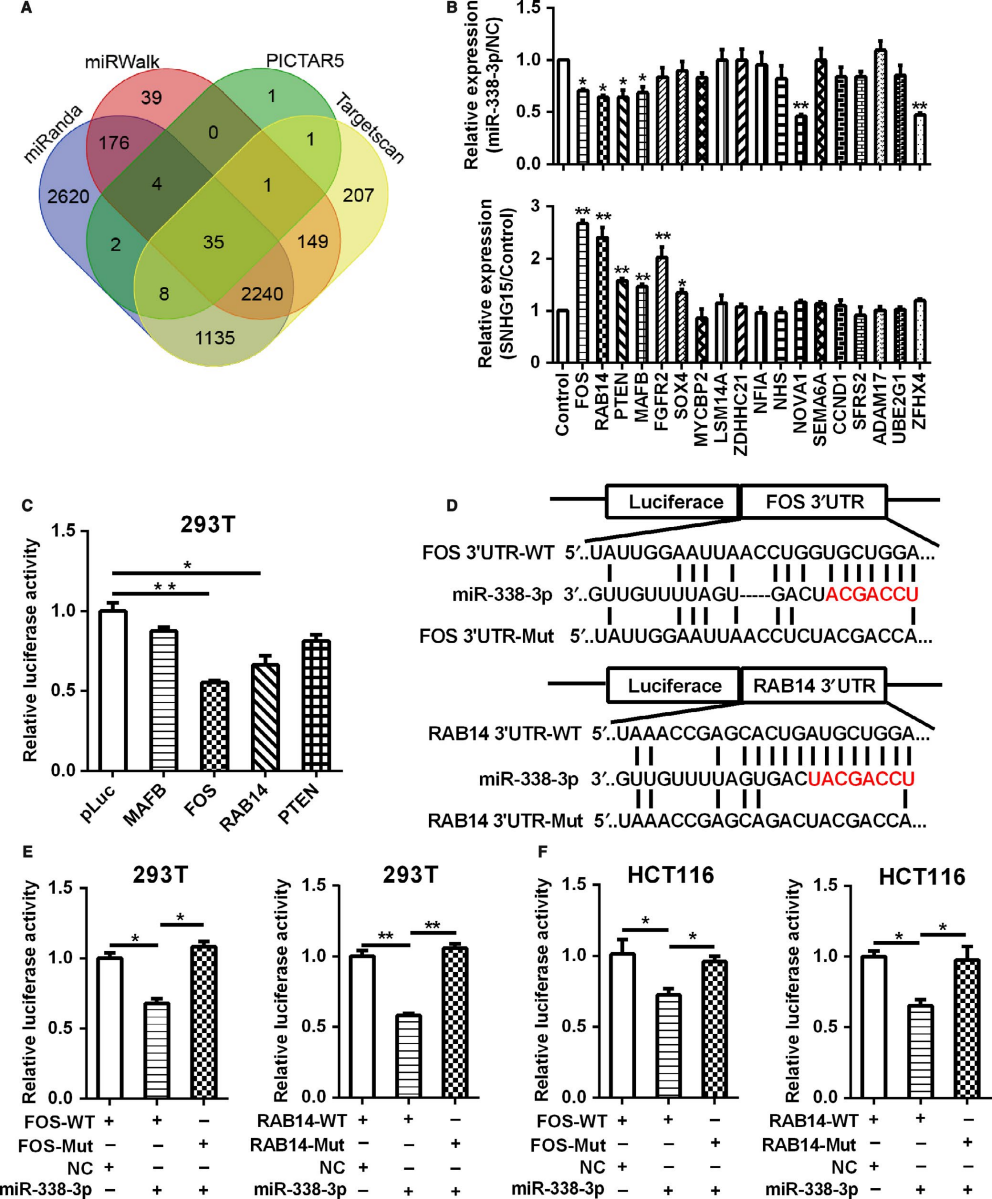
FOS and RAB14 were target genes of miR‐338‐3p. A, The potential target genes of miR‐338‐3p were predicted by the databases miRanda, miRWalk, PICTAR5 and Targetscan. B, The relative expression of predicted target genes of miR‐338‐3p in SNHG15 or miR‐338‐3p overexpressed LoVo cells. C, The potential target genes of miR‐338‐3p were verified by the dual luciferase assay. D, The wild‐type or mutant 3′UTR sequence of FOS and RAB14 binding with miR‐338‐3p were cloned into the pLuc vector separately. E‐F, The luciferase activity of pLuc‐FOS/RAB14‐WT or pLuc‐FOS/RAB14‐mut after cotransfected with miR‐338‐3p was determined by the dual luciferase assay in 293T cells (E) and HCT116 cells (F). *P < 0.05, **P < 0.01

Recent evidence suggests that several CRC‐related lncRNAs may also regulate gene expression by binding to specific miRNAs and consequently preventing these miRNAs from inhibiting their target mRNAs. In order to get the downstream genes of SNHG15/miR‐338‐3p axis, we firstly searched for the potential targets of miR‐338‐3p using the databases miRanda, miRWalk, PICTAR5 and Targetscan, and got 35 copredicted target genes at last (Figure 5A). We chose 18 oncogenic genes that are overexpressed in human tumors for further verification. Next, we examined the effect of SNHG15 and miR‐338‐3p overexpression on the expression levels of those candidate genes. The results showed that the expression of FOS, RAB14, MAFB and PTEN were up‐regulated in SNHG15 overexpressed CRC cells, and were down‐regulated in miR‐339‐3p overexpressed CRC cells. (Figure 5B), which suggest that these 4 genes were the potential targets of SNHG15/miR‐338‐3p axis. We further verified whether these 4 genes were targets of miR‐338‐3p using luciferase assays, the data showed that the luciferase activity of FOS or RAB14 3′UTR‐WT plasmid was significantly inhibited after overexpression of miR‐338‐3p (Figure 5C). Whereas ectopic expression of miR‐338‐3p could not reduce the luciferase activity of FOS or RAB14 3′UTR‐Mut plasmid (Figure 5D‐F), suggesting that the binding of FOS or RAB14 with miR‐338‐3p was sequence‐dependent. These results demonstrated that FOS and RAB14 were the direct targets of miR‐338‐3p.

### 3.5 SNHG15 plays tumor‐promoting role in CRC by regulating FOS and RAB14

**Figure 6:**
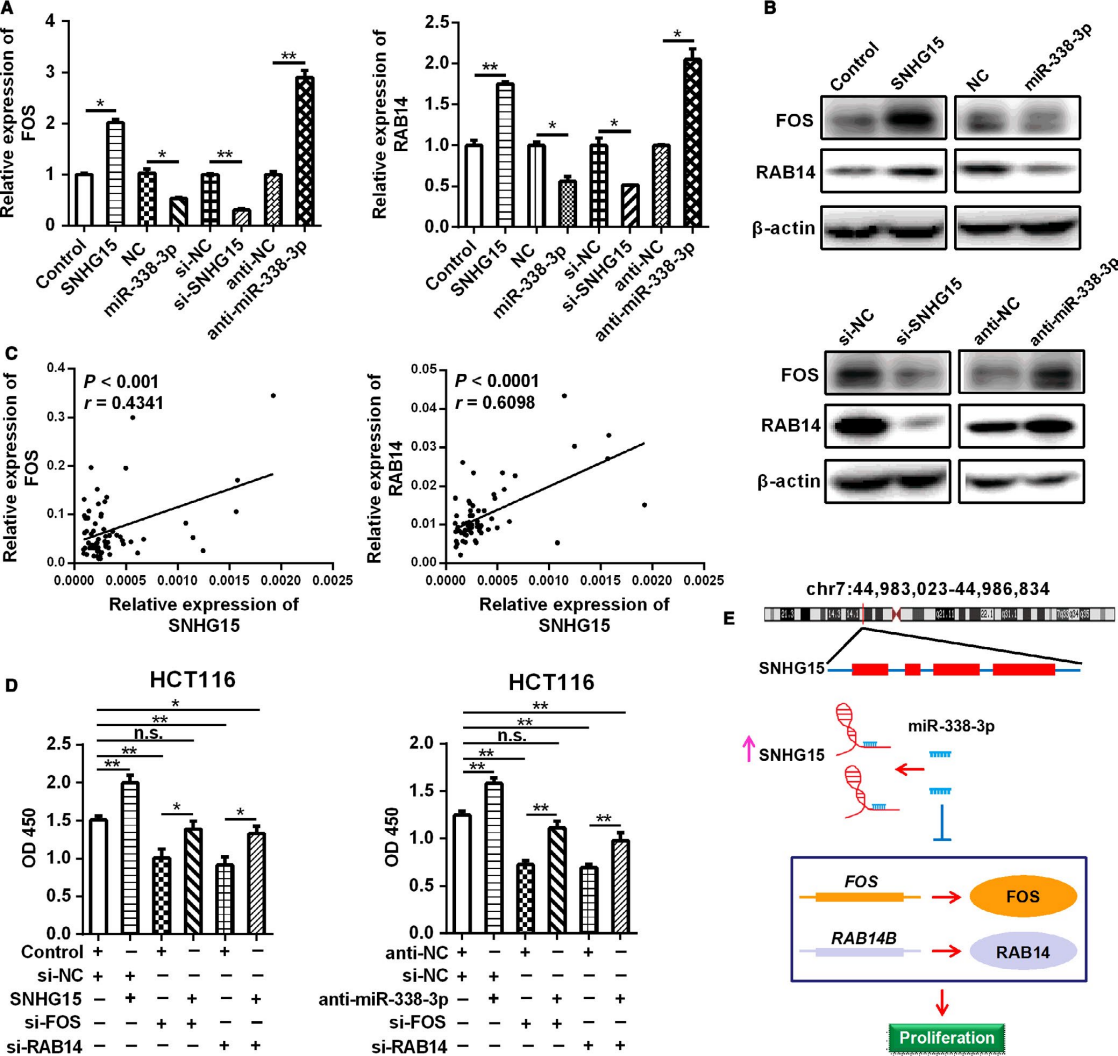
SNHG15 played tumor‐promoting role in CRC by regulating FOS and RAB14. A‐B, The changes of mRNA and protein level of FOS/RAB14 after SNHG15/miR‐338‐3p overexpression or down‐regulation were examined by qRT‐PCR and Western blot. C, Bivariate correlation analysis of the relationship between SNHG15 expression and FOS or RAB14 level, n = 64. D, The increased cell viability in SNHG15 overexpressed CRC cells was abolished by FOS or RAB14 knockdown. The increased cell viability in miR‐338‐3p‐silenced CRC cells was also abolished by FOS or RAB14 knockdown. *P < 0.05, **P < 0.01

It has been confirmed that SNHG15 was the ceRNA of miR‐338‐3p, we then speculated whether SNHG15 promote cell proliferation by competing with miR‐338‐3p and restoring the expression of FOS and RAB14. We firstly examined the mRNA and protein levels of FOS/RAB14 after up‐regulation or down‐regulation of SNHG15 and miR‐338‐3p in CRC cells. The results showed that overexpression of SNHG15 or down‐regulation of miR‐338‐3p promoted both the mRNA and protein levels of FOS and RAB14, while overexpression of miR‐338‐3p or knockdown of SNHG15 inhibited the expression of FOS and RAB14 (Figure 6A and B). A correlation analysis showed that the expression of FOS and RAB14B were positively correlated with SNHG15 levels in CRC tissues (Figure 6C, FOS vs SNHG15: *P* < 0.001, *r* = 0.4341; RAB14 vs SNHG15: *P* < 0.0001, *r* = 0.6098). To investigate whether SNHG15 and miR‐338‐3p exert tumor growth promoting function by modulating FOS/RAB14, we checked the effect of FOS and RAB14 on SNHG15‐induced cell proliferation. The results showed that FOS and RAB14 knockdown blocked the cell growth induced by SNHG15 overexpression in CRC cells. Meanwhile, FOS and RAB14 knockdown also impaired the cell growth induced by miR‐338‐3p silencing (Figure 6D). Collectively, these data indicated that the cell proliferation promoting function of FOS and RAB14 were partly regulated by SNHG15/miR‐338‐3p (Figure 6E).

## 4 DISCUSSION

In this study, we found that SNHG15 was significantly up‐regulated in CRC tissues and associated with poor survival. Functionally, SNHG15 could promote cell proliferation and inhibit cell apoptosis in CRC cells. Moreover, we further revealed that SNHG15 executed tumor‐promoting function by sponging miR‐338‐3p and increasing the expression and activities of FOS and RAB14 for the first time. These data suggest that SNHG15 functions as an oncogene in CRC, and can be used as a potential prognostic biomarker and therapeutic target for CRC.

Recent evidences have revealed that lncRNAs are frequently deregulated and play vital roles in many human cancers. Our previous studies revealed a variety of CRC related lncRNAs, which are involved in cancer promoting.^14^, ^15^, ^16^, ^17^ In addition to these lncRNAs, we also discovered a potential oncogenic lncRNA, SNHG15, in CRC. SNHG15 was initially found to be overexpressed in GC and could promote cell proliferation and invasion of GC.^19^ SNHG15 was subsequently confirmed to be up‐regulated and worked as an oncogene in hepatocellular carcinoma, osteosarcoma, pancreatic cancer, and breast cancer.^21^, ^23^, ^25^, ^26^ We also found that SNHG15 was obviously up‐regulated in CRC tissues compared with paired NCTs and predicted poor prognosis of CRC patients. Although SNHG15 has been shown to be overexpressed in colon cancer through the analysis of TCGA database in Jiang's research,^27^ we examined the expression of SNHG15 in CRC tumor specimens and identified SNHG15 was significantly up‐regulated in CRC. SNHG15 was also confirmed to be overexpressed in CRC tissues in Huang's research recently, but they did not check the effect of SNHG15 on cell function of CRC.^28^ We did not only prove that SNHG15 is highly expressed in CRC, but also found that it could promote cell proliferation of CRC in vitro and in vivo. All the results in this research suggest that SNHG15 is an important oncogenic lncRNA in CRC.

LncRNA has various functions, including chromatin remodeling, sponging, scaffolding, transcriptional activation, and so on. Most cytoplasmic lncRNAs play important roles in the regulation of gene expression by acting as ceRNAs. Chen et al found that lncRNA‐UICLM promote metastasis of CRC by acting as a ceRNA of miR‐215.^29^ Previous studies in our group found that lncRNA UCA1 and LINC00152 both function as ceRNAs to promote CRC cell proliferation and chemoresistance.^15^, ^16^ SNHG15 has already been confirmed to promote CDK14 expression by sponging miR‐486 in NSCLC.^30^ It could also promote breast cancer proliferation and metastasis through binding with miR‐211‐3p.^21^ These suggest that ceRNA is one of the most important mechanisms of SNHG15 in cancer. So we further searched and verified the miRNAs that SNHG15 could sponge in CRC, the results of dual luciferase reporter assay suggested that SNHG15 could bind with miR‐338‐3p and inhibit its function. Previous studies have demonstrated that miR‐338‐3p could function as a tumor suppressor in GC, NSCLC and CRC.^31^, ^32^, ^33^ We also confirmed the anti‐carcinogenic effect of miR‐338‐3p in CRC in this study, and the function of miR‐338‐3p is inhibited after sponged by SNHG15.

We further searched for the targets of the axis of SNHG15/miR‐338‐3p, and found FOS and RAB14 were the downstream genes of SNHG15/miR‐338‐3p axis by qPCR and dual luciferase reporter assay. FOS is one member of AP‐1 protein family transcription factors and overexpressed in lots of human cancers.^34^ FOS plays an important role in tumor cell proliferation and metastasis.^35^ RAB14 is highly expression in multiple human cancers, including CRC. RAB14 also functions as an oncogene by promoting tumorigenesis and metastasis.^36^ In our study, we discovered that FOS and RAB14 were the new target genes of miR‐338‐3p, and the expression of FOS and RAB14 were both regulated by SNHG15 and miR‐338‐3p. Subsequent functional experiments further confirmed that SNHG15 regulates CRC development and progression by competitively sponging miR‐338‐3p and restoring the activity of FOS and RAB14.

In summary, our study highlighted that SNHG15 was up‐regulated and exerted an oncogenic role in cell proliferation of CRC. We also demonstrated that SNHG15 served as a ceRNA and reversed the activity of miR‐338‐3p, thus facilitating CRC tumorigenesis by enhancing the expression of FOS and RAB14. Taken together, all these results suggest that the SNHG15/miR‐338‐3p/FOS ‐RAB14 axis may serve as a novel therapeutic target for CRC.

## ACKNOWLEDGMENT

This work was supported by the National Natural Science Foundation of China (81672328, 81802469, 81772636 and 81802462), Natural Science Foundation of Jiangsu Province (BK20150004, BK20151108 and BK20180618), Fundamental Research Funds for the Central Universities (NOJUSRP51619B and JUSRP51710A), Medical Key Professionals Program of Jiangsu Province (AF052141), Medical Innovation Team Program of Wuxi (CXTP003), Hospital Management Centre of Wuxi (YGZXZ1401), National First‐class Discipline Program of Food Science and Technology (JUFSTR20180101) and Project of the Wuxi Health and Family Planning Commission (Z201806).

## CONFLICT OF INTEREST

The authors declare no conflict interest.
