# Supplementary material for: LncRNA‐SNHG15 enhances cell proliferation in colorectal cancer by inhibiting miR‐338‐3p
Source: Cancer Med. 2019 Apr 3;8(5):2404–13. doi: 10.1002/cam4.2105 (PMC6536931; doi:10.1002/cam4.2105)
Supplement: Supplementary file 1 [file CAM4-8-2404-s001.doc]

**Supplementary Tables**

**Supplementary Table S1**. Clinicopathologic features of CRC patients

| **Characteristics** | **Colorectal cancer^a^**  **(n=113)** | **Colorectal cancer^b^**  **(n=90)** |
| --- | --- | --- |
| Ages (years) |  |  |
| < 60 | 63 | 45 |
| ≥ 60 | 50 | 45 |
| Gender |  |  |
| Male | 65 | 52 |
| Female | 48 | 38 |
| Tumor size (cm) |  |  |
| < 5 | 68 | 43 |
| ≥ 5 | 45 | 47 |
| Location |  |  |
| Colon | 61 | 50 |
| Rectum | 52 | 40 |
| Differentiation |  |  |
| Well and moderately | 92 | 81 |
| Poorly | 21 | 9 |
| Invasion depth |  |  |
| T1 | 4 | 3 |
| T2 | 18 | 20 |
| T3 | 26 | 43 |
| T4 | 65 | 24 |
| Lymphatic invasion |  |  |
| Absent | 52 | 63 |
| Present | 61 | 27 |
| Distant metastasis |  |  |
| Absent | 99 | 87 |
| Present | 14 | 3 |
| Tumor stage |  |  |
| I | 16 | 21 |
| II | 30 | 41 |
| III | 54 | 25 |
| IV | 13 | 3 |

^a^: 113 CRC tissues and their paired adjacent NCTs were collected at Affiliated Hospital of Jiangnan University. ^b^: 90 CRC tissues and their paired adjacent NCTs were collected at Fudan University Shanghai Cancer Center.

**Supplementary Table S2**. Primer sequences

| Gene | Forward primer | Reverse primer |
| --- | --- | --- |
| SNHG15 | GGTGACGGTCTCAAAGTGGA | GCCTCCCAGTTTCATGGACA |
| FGFR2 | GGAAAGTGTGGTCCCATCTGA | TCCAGGTGGTACGTGTGATTG |
| MYCBP2 | GGGGACGGATTCTACCCAG | ATTGAGCGCAGCGGTATAAAT |
| FOS | GGGGCAAGGTGGAACAGTTA | AGTTGGTCTGTCTCCGCTTG |
| LSM14A | CACAGGCGAGCTGAAGTACA | CACAGGCGAGCTGAAGTACA |
| ZDHHC21 | CATGGGCTTGATTGTCTTTGT | ACGTGATTGGCAAAGTGGTAG |
| NR3C2 | CATCATGAAAGTTTTGCTGCTACT | TCTTTGATGTAATTTGTCCTCATTTCA |
| NFIA | TGGCCAAGTTACGGAAAGAT | GCGCTCGCCATCAGTACT |
| NHS | GAGACCCAAGGAAATGTGGA | ATGTCCCCGGAATCTTTTCT |
| NOVA1 | TACTGAGCGAGTGTGCTTGAT | GTCTGGGGTTGTAGAATGCTG |
| RAB14 | GACAGATGCAAGGAATCTCACC | GCTTCGAGGAACAATAAGCCAT |
| SEMA6A | ACAATTCCTTTGTGGCACTGAA | TCTTGAGCCGTGGAATCTGA |
| cyclin D1 | CCTGTCCTACTACCGCCTCA | TCCTCCTCTTCCTCCTCCTC |
| SFRS2 | GCTGAGGACGCTATGGATG | GCGGCTGTGGTGTGAGTC |
| SOX4 | ACAGCGACAAGATCCCTTTC | CGGACTTCACCTTCTTCCTG |
| ADAM17 | AGAGCTGACCCAGATCCCAT | TACTCTCTTCCCCTCTGCCC |
| UBE2G1 | GCTGGCAGACCCTAATGGAG | TTCTTACACAGCGGGCAACT |
| ZFHX4 | GGAGAACTGTGGGCAGAGAG | AGGTAAGGTCCGCTTTGGTT |
| MAFB | GTGCAGGTATAAACGCGTCC | CACCTCCTGCTTAAGCTGCTC |
| β-actin | AGTGTGACGTGGACATCCGCAAAG | ATCCACATCTGCTGGAAGGTGGAC |

**Supplementary Figures**

**Supplementary Figure S1**

**
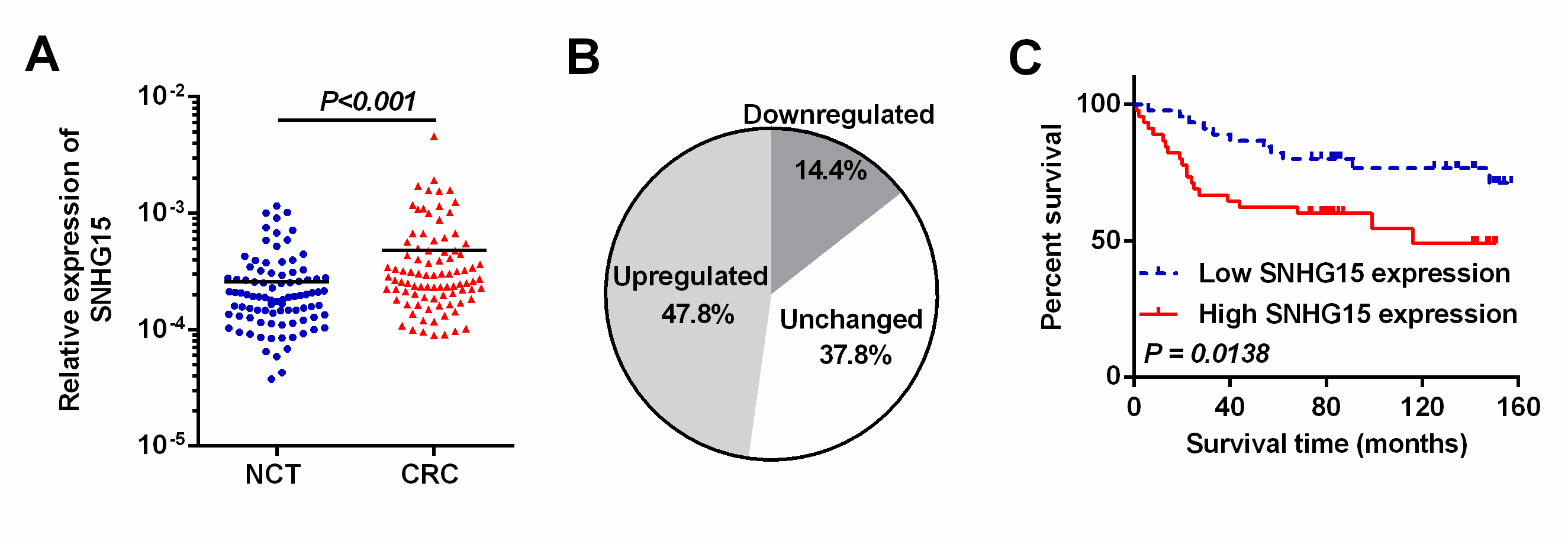
**

**Figure S1.** SNHG15 is upregulated in an independent CRC cohort. A, The expression level of SNHG15 was analyzed in an independent CRC cohort by qRT-PCR, *P* < 0.001. B, FEZF1-AS1 was upregulated in 47.8 % of 90 paired CRC tissues. C, Survival analysis of another CRC cohort which are grouped by the expression of SNHG15. *P* = 0.0138.

**Supplementary Figure S2**

**
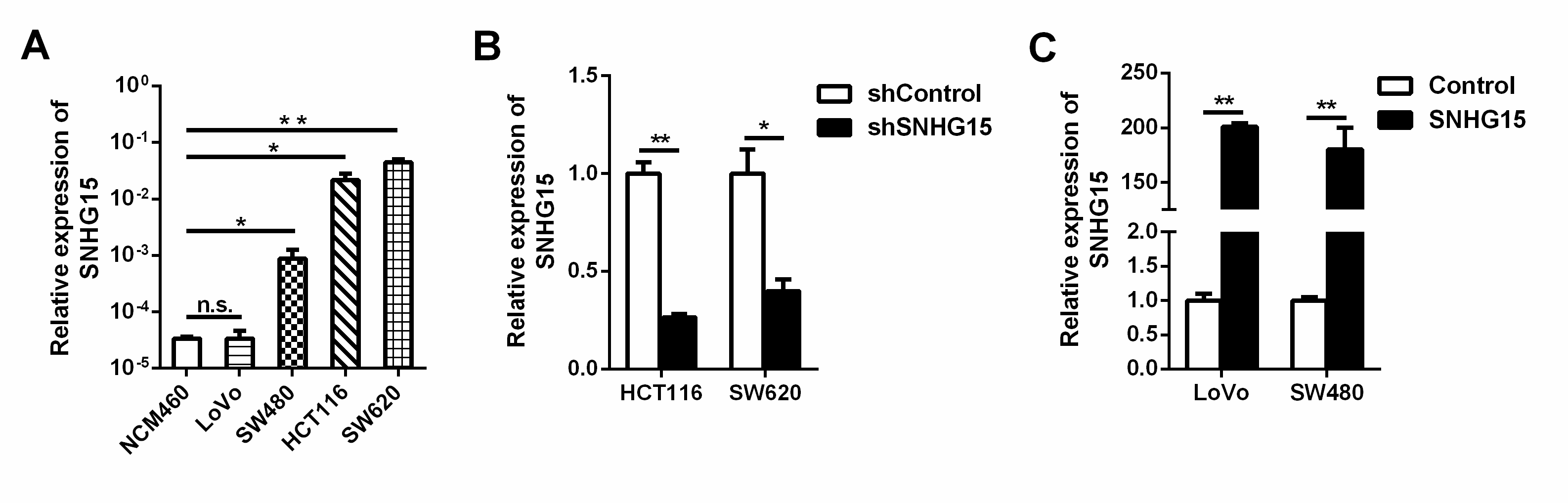
**

**Figure S2.** The effects of SNHG15 knockdown and overexpression were verified in CRC cells. A, Relative expression of SNHG15 in four CRC cell lines was examined by qRT-PCR. B-C, The efficacy of SNHG15 knockdown or over-expression in CRC cell lines was examined by qRT-PCR. The expression of SNHG15 was inhibited in HCT116 and SW620 cells; and was over-expressed in LoVo and SW480 cells. * *P* < 0.05, ** *P* < 0.01.

**Supplementary Figure S3**

**
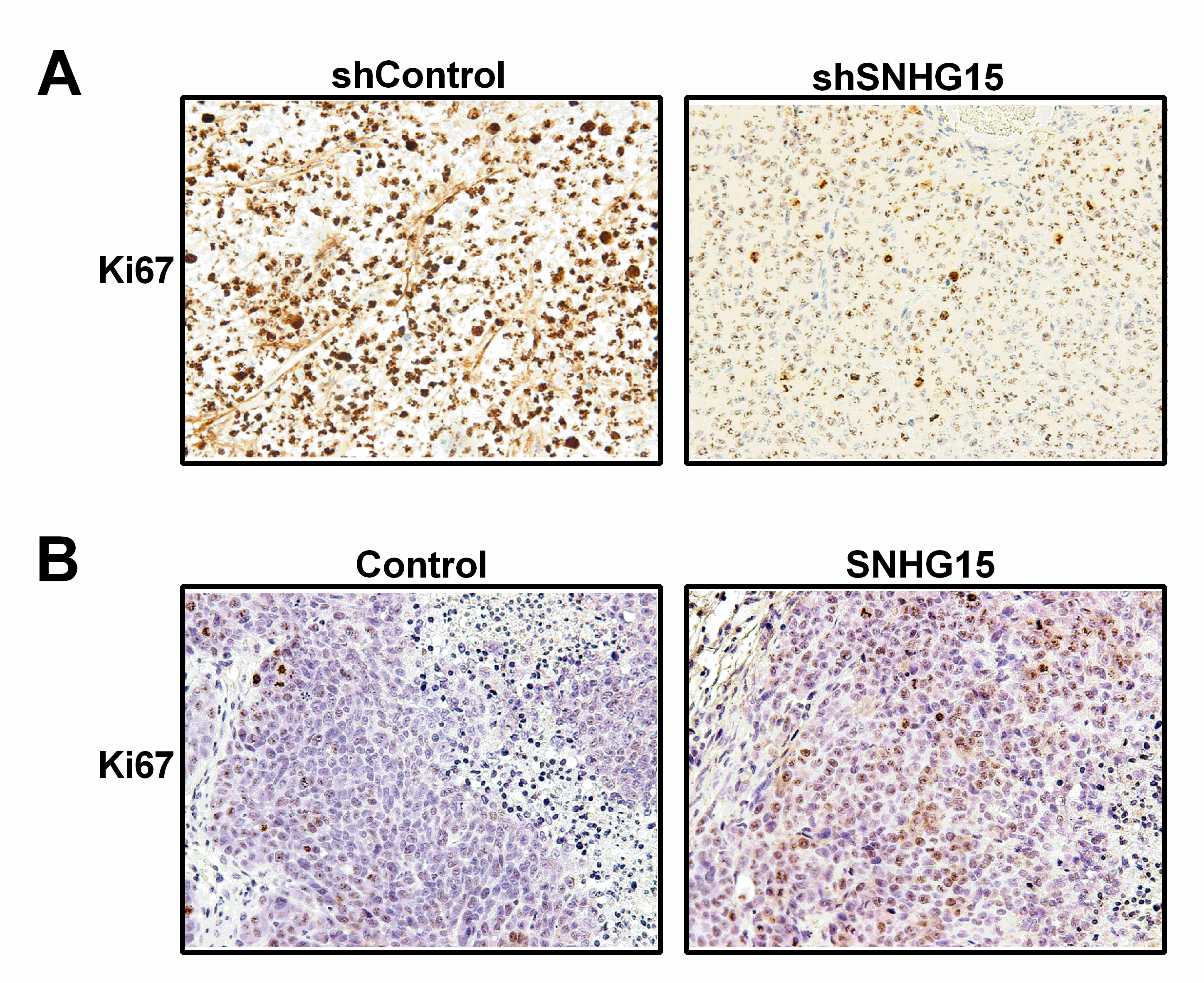
**

**Figure S3.** Xenograft tumors were analyzed for Ki67 expression using immunohistochemistry. A, The expression of Ki67 was downregulated in the SNHG15-depleted tumors. B, The expression of Ki67 was upregulated in the SNHG15-overexpressing tumors.

**Supplementary Figure S4**

**
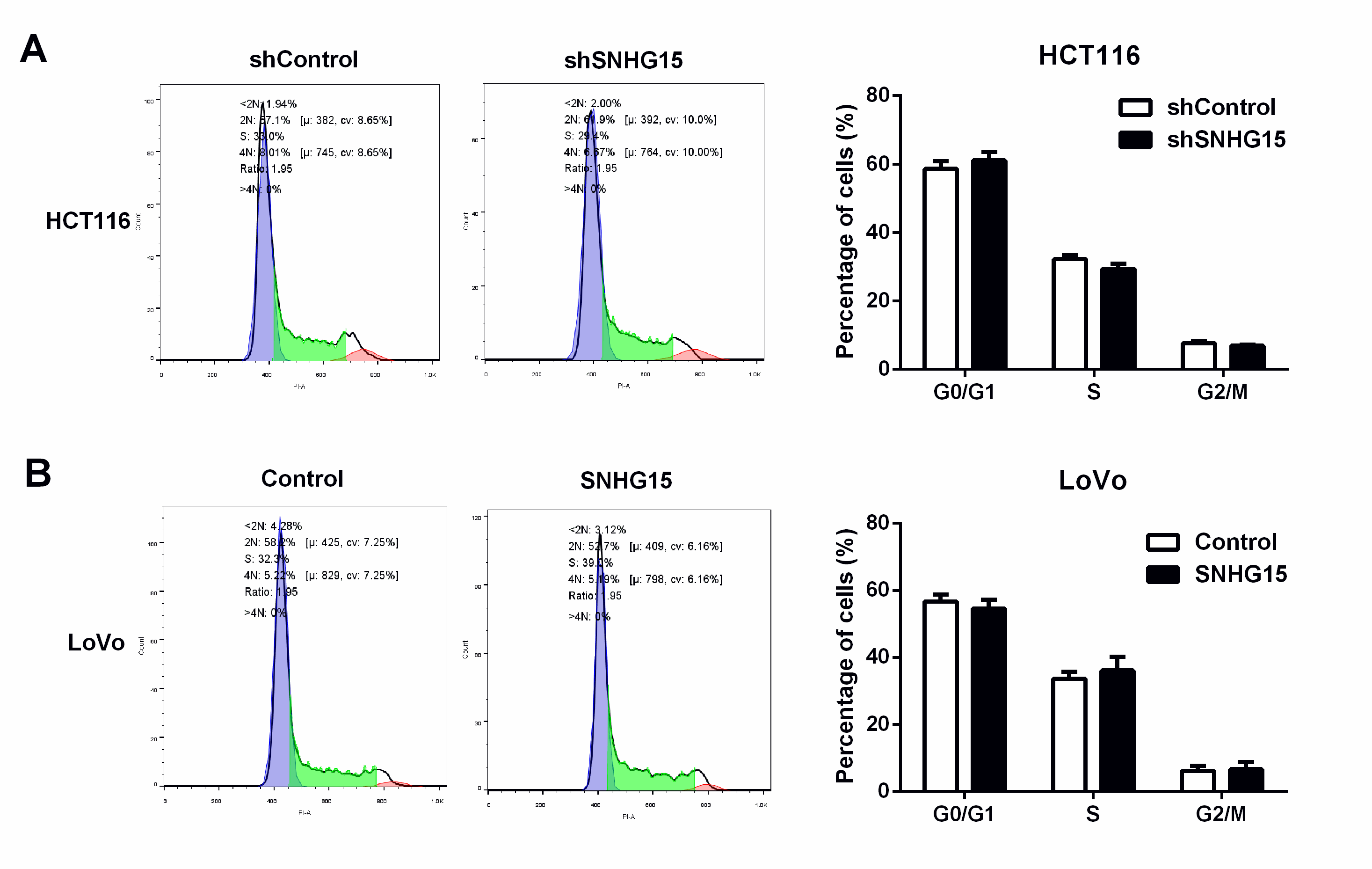
**

**Figure S4.** The effect of SNHG15 on cell cycle distribution was determined by flow cytometry. A, Knockdown of SNHG15 had no effect on cell cycle distribution in HCT116 cells. B, Over-expression of SNHG15 had no effect on cell cycle distribution in LoVo cells.
